# Genetic Diversity and Recombination in the Plant Pathogen *Sclerotinia sclerotiorum* Detected in Sri Lanka

**DOI:** 10.3390/pathogens9040306

**Published:** 2020-04-22

**Authors:** Thirega Mahalingam, Weidong Chen, Chandima Shashikala Rajapakse, Kandangamuwa Pathirannahalage Somachandra, Renuka Nilmini Attanayake

**Affiliations:** 1 Department of Plant and Molecular Biology, University of Kelaniya, Kelaniya 11600, Sri Lanka; m.thirega52lingam@gmail.com; 2 United States Department of Agriculture-Agriculture Research Service (USDA-ARS), Grain Legume Genetics and Physiology Research Unit, Washington State University, Pullman, WA 99164, USA; weidong.chen@usda.gov; 3 Department of Chemistry, University of Kelaniya, Kelaniya 11600, Sri Lanka; shashikala@kln.ac.lk; 4 Regional Agricultural Research and Development Centre, Bandarawela 90100, Sri Lanka; kpsomachandra@gmail.com

**Keywords:** *Sclerotinia sclerotiorum*, mycelial compatibility grouping, recombination, genetic structure, genetic diversity, cabbage

## Abstract

*Sclerotinia sclerotiorum* is an important fungal pathogen on many economically important crops including cabbage worldwide. Even though population structure and genetic diversity of *S. sclerotiorum* is well studied in temperate climatic conditions, only a few studies have been conducted in tropical countries. It is also not clear whether the populations are clonal or recombining in the tropics. In filling this information gap, 47 isolates of *S. sclerotiorum* were collected from commercial cabbage (*Brassica oleracea* L.) fields in Nuwara Eliya district of Sri Lanka, where the disease has been previously reported. All the isolates were subjected to genetic diversity study using mycelial compatibility grouping and microsatellite markers. Fourteen mycelial compatibility groups (MCGs) and 23 multilocus haplotypes (MLHs) were recorded. Mean expected heterozygosity of the population was 0.56. MLHs were weakly correlated with MCGs. Population genetic structure analysis and principal coordinates identified three genetic clusters. Genetic recombination was inferred within each genetic cluster when isolates were subjected to clone correction. There was evidence of multiple infections on single plant as detected by the presence of more than one MCG on each cabbage plant. However, multiple infections did not increase the disease severity in detached leaf assay. We found high genetic diversity and recombination of *S. sclerotiorum* population in a tropical country, Sri Lanka. Importance of detecting genetic structure when inferring recombination was also highlighted.

## 1. Introduction

*Sclerotinia sclerotiorum* is a cosmopolitan filamentous fungus causing infections on more than 600 plant species including important vegetables such as tomato, bean, carrot and cabbage [1]. No complete resistant cultivars are available for many host crops including cabbage [2] and fungicide application is a widely used management practice. High levels of variation in fungicide insensitivities among *S. sclerotiorum* isolates have been reported in the USA [3], China [4], Iran [5], Sri Lanka [6], and other European countries [7]. However, the selected fungicide should be effective against all the genotypes and most importantly, the prevalent genotype of a population to achieve the best disease management. Therefore, understanding genetic diversity and structure of the pathogen population is important in devising disease management strategies. 

Population diversity and genetic structure of *S. sclerotiorum* have been well studied around the world describing clonality based on the self-fertility and predominant asexual reproduction by sclerotia [8,9,10,11,12]. In contrast, recent studies conducted in Brazil [13], China [4], Iran [5], New Zealand [14], the USA [3,4,11], and UK [7] reported genetic recombination and mixed population structures of *S. sclerotiorum*. However, except for few studies conducted in tropical countries [15,16], almost all the studies have been conducted using pathogen samples from temperate and sub-tropical countries. Gomes et al. [13] and Litholdo Júnior et al. [17] reported high genetic diversity and clonal as well as recombining populations of *S. sclerotiorum* in a tropical country, Brazil. Lehner et al. [16] reported that there is no difference in genetic diversity in tropical and temperate conditions. Under this circumstance, it is clear that studies conducted in tropics are insufficient and therefore, the current study aimed in filling this information gap.

In Sri Lanka, *S. sclerotiorum* on cabbage (*Brassica oleracea* L.) was first reported in 2014 [18] and since then, the disease has been detected in commercial cabbage fields from time to time. However, except only two studies on pathogen phenotypic diversity [19] and anaerobic soil disinfestation based disease management [6], no population genetics studies of *S. sclerotiorum* have been conducted in Sri Lanka. Hence, Sri Lanka provides an excellent location to study the genetic diversity and population structure of *S. sclerotiorum* under tropical climate.

Under conducive conditions and high level of pathogen population diversity, multiple infections per single host plant are often reported [20,21,22,23]. When a host is infected by different species/genotypes of a pathogen, inhibition, co-survival or generation of new genotypes via recombination is possible ultimately compromising the host fitness [20,21,22,23]. Multiple genotypes of a pathogen species infecting a single host plant (here after referred as multiple infections) have been reported in; *Cryphonectria parasitica* on chestnut [24], *Fusarium* spp. on maize [25], *Mycosphaerella graminicola* on wheat [26], and *Microbotryum violaceum* on *Silene latifolia* [27]. To the best of our knowledge, except the studies conducted in the USA [28] and Australia [29] on canola, no other reports available on multiple infections of *S. sclerotiorum* on other hosts. It is also not known whether multiple infections by *S. sclerotiorum* can be detected under tropical climate or not. Therefore, the objectives of this study are to assess the genetic diversity and population structure of *S. sclerotiorum* as a model pathogen of cabbages cultivated in Sri Lanka, to determine whether the pathogen population is recombining or clonal under tropical climate, to determine whether multiple infections of *S. sclerotiorum* are found on cabbage in Sri Lanka and to evaluate whether the disease severity is affected by the infection of more than one genotype of *S. sclerotiorum* in cabbage.

## 2. Results

### 2.1. Mycelial Compatibility Group (MCG) Diversity

The isolates were grouped based on mycelial compatibility exhibited in paired MCG tests. Isolates that showed mycelial compatibility were grouped into one MCG and that showed incompatibility were grouped into different MGCs. Among the 47 isolates of *S. sclerotiorum*, 14 MCGs were detected. The most common MCG contained 14 isolates. Ten MCGs were represented by single isolates. On average there were 3.4 isolates per MCG in the population. Origin of the *S. sclerotiorum* isolates and their MCG assignments are shown in the Table 1.

### 2.2. Multilocus Haplotype (MLH) Diversity

Eight microsatellite loci of *S. sclerotiorum* were polymorphic in the present study and used in population scale genotyping. For every isolate, a single allele per locus was amplified as expected for a haploid organism like *S. sclerotiorum*. In the population of *S. sclerotiorum,* there were two to nine different alleles, averaging 4.8 alleles per locus (Table 2). Allele size variation was due to the differences in number of microsatellite repeat motifs in each locus. The total expected heterozygosity (*He*) for each of the eight loci ranged from 0.08 for locus 55-4 to 0.83 for locus 114-4 (Table 2). A unique combination of the alleles of the eight microsatellite loci was identified for each isolate and the isolates that shared the same combination of the alleles were grouped into a multilocus haplotype (MLH) (Table 1). There were 23 MLHs among the 47 isolates. Two MLHs consisted of six isolates each. Fifteen haplotypes or 31.9% of the population is represented by single isolates and on average there were 2.04 isolates per MLH. Even though isolates of the same MLH generally belonged to the same MCG, there were some discrepancies. For example, the third MLH consisted of isolates belonged to two MCGs, fourth MLH consisted of isolates belonged to three MCGs and sixth MLH consisted of isolates of three MCGs (Table 1). A comparison of genetic diversity of the current population with Attanayake et al. [30], in which the same microsatellite markers were used is shown in the Table 3.

**Figure 1:**
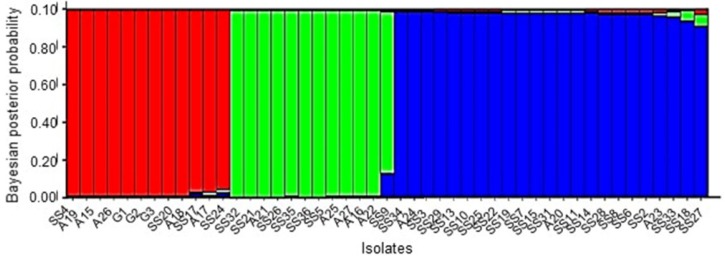
Bar plot from STRUCTURE analysis when K = 3, showing the *Sclerotinia sclerotiorum* isolates assigned into three genetic clusters depicted in three colors. Y axis shows the Bayesian posterior probability of population assignment and each bar represents each isolate.

**Figure 2:**
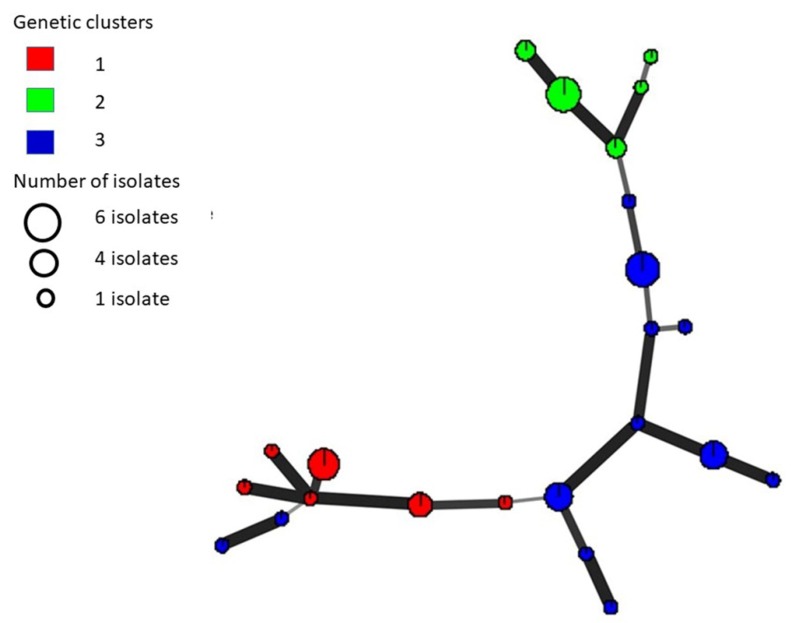
Minimum spanning network with unique haplotype indicated by a circle and circle size is proportional to the number of isolates. Each haplotype is color coded as per STRUCTURE output and line thickness is proportionate to Bruvo’s distance. Line lengths are arbitrary.

In STRUCTURE analysis, log likelihood values (Ln P(D)) increased from K = 1 to 3 and then plateaued with a sharp increase in variance indicating that the pathogen population is best explained by three distinct genetic clusters. Mean Ln P(D) value at K = 3 was −196 ± 37.4 and population clustering is shown the Figure 1. The biggest genetic cluster (blue) comprised of 23 isolates from 12 haplotypes while the other two clusters (red and green) contained 12 isolates each from six and five haplotypes respectively. Principal component analysis (PCoA) of microsatellite data also illustrated the presence of three clusters. The first three principal components explained 31.80%, 25.17%, and 12.15% of the total genetic variation respectively. Minimum spanning network (MSN) generated using Bruvo’s distance among isolates also showed the separation of three clusters marked in three colors (Figure 2). The thickness of the lines between the dots indicates the means of relatedness among individuals.

### 2.3. Detection of Recombination

When the whole population was subjected to the multilocus linkage disequilibrium analysis, the null hypothesis of random association was rejected even after clone correction. However, when *I_A_* test was performed on three subpopulations inferred from STRUCTURE, two clusters failed to reject the null hypothesis of random association. After clone correction, all three subpopulations failed to reject the null hypothesis of random association (Table 4).

When pairwise linkage disequilibrium analysis was performed for the eight microsatellite loci, 15 out of 28 possible pair-wise comparisons displayed significant (*p* < 0.001) linkage disequilibrium. However, random association (linkage equilibrium) between all the pairs of alleles were detected when the analysis was conducted with clone-corrected data (Table 5).

### 2.4. Distribution of Mating Type Genes

As in other homothallic fungal species, *S. sclerotiorum* carries both *MAT 1-1* and *MAT 1-2* idiomorphs within its genome. Among a sub-population of selected 40 isolates, 52% of isolates had both *MAT* idiomorphs and the remaining 48% of the isolates amplified only *MAT 1-2*. The lack of amplification of *MAT 1-1* idiomorph was due to an inversion of the gene [32] and named as inversion plus isolates whereas the rest of the isolates were named as inversion minus. Chi-square test (*p* < 0.05) showed distribution probability of the inversion minus isolates and inversion plus isolates in the population were equal (1:1).

### 2.5. Detection of Multiple Infections per Cabbage Head

**Figure 3:**
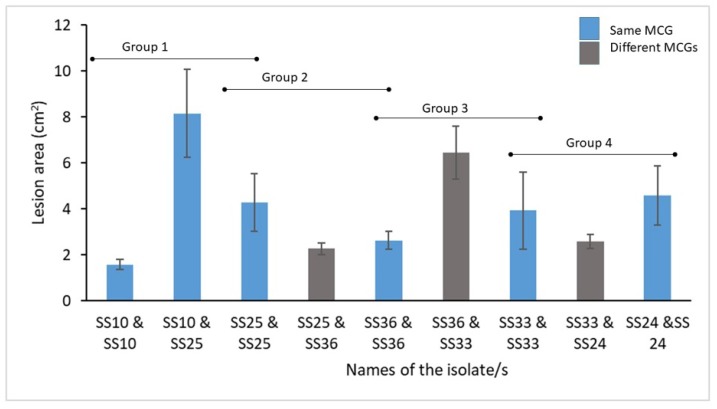
Bar graph showing the variation in lesion areas (cm^2^) of cabbage leaves inoculated with one or two different isolates of *Sclerotinia sclerotiorum*. Isolates of the same mycelial compatibility group (MCG) are shown in blue color whereas the isolates of different MCGs are shown in ash color. Whiskers indicate one standard error of the mean. X axis shows the names of *S. sclerotiorum* isolates.

All the 20 isolates obtained from two cabbage heads in the first infection locus were compatible among each other (within the head as well as between the two heads) indicating the presence of a single MCG. Similarly, all of the 30 isolates collected from three cabbage heads in the second infection focus were also compatible among each other. However, there were two MCGs in the third infection focus, which was consisted of 30 isolates collected from three symptomatic cabbage heads. Two isolates were belonged to a single MCG whereas the rest of the 28 isolates belonged to a different MCG. Attempts were made to test whether multiple infections affect the disease severity in detached leaf assay. Figure 3 shows the variation in lesion areas when one MCG or two MCGs were co-inoculated on detached cabbage leaves.

One-way ANOVA for four groups (Group 1: isolates SS10 and SS25; Group 2: isolates SS25 and SS36; Group 3: isolates SS36 and SS33; Group 4: isolate SS33 and SS24 as shown in Figure 3) showed that there was no significant difference in lesion areas within a group. *p* values for each group from 1 to 4 were 0.56, 0.21, 0.47, and 0.81 respectively. Tukey’s pairwise comparison yielded that there was no significant difference in lesion areas between all possible pairs within each group.

## 3. Discussion

Population genetic structure of *S. sclerotiorum* is well studied around the world, mainly in temperate regions of the world and reported clonality [8,9,10,33] as well as mixed population structures [3,4,11,34]. No much information is available for the tropical countries. This study provides new information on genetic diversity, population structure and recombination of the pathogen population in a tropical country, Sri Lanka, for the first time. Since the disease is frequently detected in Nuwara Eliya district where extensive vegetable cultivation is taking place and no severe disease pressure has been reported in the low land cabbage cultivating areas (Personal communication, K.P. Somachandra, Department of Agriculture), this study was concentrated on the samples collected from several locations of Nuwara Eliya district. All the isolates used in the population analysis were confirmed to be *S. sclerotiorum* based on the typical morphological characters on potato dextrose agar (PDA), sclerotial size and successful amplification of *MAT* loci and *S. sclerotiorum* specific microsatellite markers.

Based on MCG and microsatellite data, relatively a high level of gene and genotypic diversity was found in Sri Lanka. MCGs were not in complete agreement with the microsatellite haplotypes as observed in other studies [3,30]. To the best of our knowledge, no previous studies used microsatellite makers for the population scale analysis of *S. sclerotiorum* on cabbage host. Therefore, no study is available for direct comparison of genetic diversity with the current study. Further, a comparison of genetic diversity of *S. sclerotiorum* among different studies is also not possible due to the differences in hosts and different kinds of marker systems or microsatellite markers used. However, the same eight microsatellite markers used in this study as the study conducted by Attanayake et al. [30], which made possible to compare the genetic diversity measurements (Table 3). Even though sample sizes and the hosts were different, in general Sri Lankan population had a higher gene diversity. However, genotypic diversity was relatively lower than that of the Attanayake et al. [30]. The average gene diversity of microsatellite loci (*H¬e* = 0.56) was analogous to those reported for non-clonal populations (*He* = 0.40–0.71) of *S. sclerotiorum* [6,35]. We observed a higher haplotype frequency (49%) than what is reported for clonal pathogen populations in the USA [36] and recombining populations in the North Central United States [34]. Three genetic clusters detected in STRUCTURE analysis were in agreement with PCoA analysis as well as with the MSN. Further, mapping of MCGs onto MSN indicated that MCGs do not give the full picture of genetic structure of a population.

Genetic recombination of *S. sclerotiorum* in Sri Lanka was evident in pairwise linkage disequilibrium analysis as well as *I_A_* test conducted within genetic populations. Similar observations have been reported on *Coccidioides immitis* [37], *Cryptococcus gattii* [38], *Phytophthora infestans* [39], *Alternaria alternate* [40] and *S. sclerotiorum* [4]. Linkage equilibrium was observed in these studies, when the *I_A_* analysis was performed on the subpopulations inferred by STRUCTURE disregarding the geographic assignment of each isolate. This infers that the individuals of a population of *S. sclerotiorum* do not recombine with individuals of other populations, but recombination could occur among individuals within genetic clusters or sub populations. Therefore, it is clear that cryptic population structure reduces the power of detecting the recombination [41]. In heterothallic fungi, mating loci carry one of two *MAT* idiomorphs; *MAT 1-1* and *MAT 1-2* as well as other related genes such as *MAT 1-1-1, MAT 1-1-2, MAT 1-2-1*. In homothallic fungi, both idiomorphs and its related genes are present in a single locus [42]. Chitrampalam et al. [32] reported inversion of mating type loci of *S. sclerotiorum* by the truncation at the 3’ end of *MAT 1-1-1* allele, producing 3.6 kb inversion of *MAT 1-2-1* and *MAT 1-2-4* mating type genes in every meiosis and such individuals were named as inversion plus. In the present study, 50% of the isolates were inversion plus. Though the mechanism of mating type loci inversion in the outcrossing process is still unclear, higher inversion rates might induce error in mating type idiomorphs possibly of preventing self-fertility and promoting outcrossing [41].

Infected cabbage plants sometimes occur in patches. Infected plants occurring adjacent to each other referred as an infection focus in the current study. Repeated discovery of the same MCG from each infection focus tells that clonal propagation is prevalent and helps spreading pathogen from one plant to the next. It was presumed that due to rapid disease spread and necrotrophic nature of the pathogen, presence of multiple genotypes per single host plant would be rare. However, similar to Sexton et al. [43] where multiple genotypes were found to infect a single canola stem, cabbage heads with multiple infections were observed in this study. This method was not able to detect multiple infections by isolates of the same MCG. However, if multilocus genotypes were used and more sampling sites were included, the discovery rate of multiple infections would have been higher. It is not known whether infections by more than a single genotype in a single host plant have a direct effect on the disease severity in *S. sclerotiorum* on cabbage. To assess this, detached leaf assay was used. We found that no significant difference in disease severity as measured by lesion area when isolates of different MCGs were inoculated together on the same detached leaf. In contrast to the current findings, Maltby and Mihail [28] reported difference in competitiveness as measured in various virulence parameters when more than single MCG is infected the same canola plant. Detached leaf assay was conducted to minimize practical difficulties of in planta assays and to make sure that the microenvironment is strictly controlled for each treatment to reduce the error component.

In conclusion, we detected high genetic diversity of *S. sclerotiorum* in Sri Lanka, providing new information from a tropical country. Genetic recombination within the subpopulations or genetic clusters was detected. Recombining populations have the potential to generate new genotypes that are better adapted to environmental variations and control measures and it would challenge the disease management practices. Multiple infections on cabbage was detected. However, there is no enough evidence to prove that multiple infections of *S. sclerotiorum* on cabbage would change the disease severity as measured in detached leaf assay.

## 4. Materials and Methods

### 4.1. Sample Collection, Establishment of Pure Cultures, and MCG Assays

**Figure 4:**
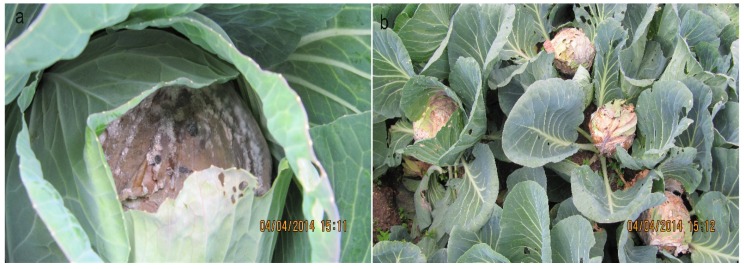
Signs and symptoms of the white mold disease on cabbage head infected by *Sclerotinia sclerotiorum.* (**a**) A cabbage plant with necrotic lesions, white mycelia and sclerotia on the top and covered with healthy leaves in the field (**b**) An infection focus showing four adjacent infected cabbage heads, three from a single raw and one on another raw.

Cabbage plants infected with *S. sclerotiorum* were easily recognized in the field with typical necrotic lesions with white color fluffy mycelia. Presence of large size sclerotia, which is a typical sign of the *S. sclerotiorum* on cabbage heads were frequently observed. Infected cabbage heads were often covered with healthy cabbage leaves as seen in the Figure 4a. A total of 60 sclerotia from naturally infected cabbage heads showing white mold symptoms (Figure 4a) were collected from 20 commercial cabbage fields located in major vegetable cultivating villages; Pattipola, Ambewela, Meepilimana and Seetha Eliya, in Nuwara Eliya district, Sri Lanka in late 2015. Each selected sampling point was at least six meters apart from another site to avoid clonal sampling [4]. Samples were transported in clean plastic bags to the laboratory. Sclerotia were washed in tap water, air dried for one day at room temperature and stored in 4 °C until use. Pure cultures of fungal isolates were obtained by surface sterilizing sclerotia with 3% sodium hypochlorite solution for 1 min, 70% ethanol for 2 min followed by three consecutive washings with sterilized distilled water. Excess water was removed from sclerotia using sterilized filter papers. Sclerotia were cut into two halves and plated onto PDA (Hardy Diagnostic, USA) amended with ampicillin (100 µg/mL) (Sigma-Aldrich, Belgium) and incubated at 23 °C for 5 days in the dark in an incubator (FOC-120i, Italy). After mycelial growth was observed from sclerotia, a single colony was obtained by hyphal tip culture, inoculated onto new PDA plates and incubated at 23 °C. Pure cultures were compared to the previously identified samples of *S. sclerotiorum* [18] for their medium sized (3–10 mm) black sclerotia produced in the periphery of the Petri dish, typical white to beige color cottony mycelia and mycelial growth patterns. Out of 60 samples, 47 pure cultures were obtained and maintained at 4 °C until further use. The rest of the samples failed to germinate on PDA plates. 

In addition to the above sampling, 10 sclerotia per cabbage head were collected from three randomly selected infection foci consisted of two to three heads in each location as shown in the Figure 4b from three different fields. Each field was approximately 20 m apart from each other. The first and second infection foci comprised of two and three adjacent infected heads located in a single raw and the third infection focus consisted of three adjacent infected heads located in two adjacent rows. Ten sclerotia were obtained from each cabbage head in each infection focus totaling 80 sclerotia and pure cultures of each isolate was obtained as described above.

Mycelial compatibility grouping of the 47 isolates was carried out as previously described [8]. Isolates were paired in all possible combinations on PDA amended with 75 µL/L of DELMEGE red food coloring (Essence Lanka Pvt. Ltd., Nugegoda, Sri Lanka) and incubated at 23 °C in the dark for 4 days. Actively growing mycelial discs (5 mm diam.) from one isolate was placed 3 cm apart from another in 9 cm diam. Petri dishes. Self-pairing was carried out for each isolate as a control. Mycelial compatibility was determined by congruent growth of two colonies with no visible death cell line. Incompatibility between isolates was determined by the presence of red barrage zone. Isolates that showed compatible reactions were grouped into the same MCG. The test was repeated for at least twice.

### 4.2. DNA Isolation, Microsatellite Genotyping, and Data Analysis

Genomic DNA was extracted from the 47 isolates using FastDNA^®^Spin Kit (MP Biomedicals, Solon, OH, USA). Eight microsatellite loci developed by Sirjusingh and Kohn [31] were used in polymerase chain reaction (PCR). PCR products were labelled with one of the four fluorophores (Vic, Pet, Ned, and Fam), multiplexed and genotyped using an ABI3730xl DNA Analyzer (Applied Bio systems, Foster City, CA, USA) at the Western regional small grain genotyping laboratory, USDA-ARS, Pullman, WA as described in Attanayake et al. [4]. GeneMarker software (Soft Genetics, LLC State College, PA, USA) was used for the fragment analysis. Each isolate was genotyped at least twice for each locus.

The STRUCTURE v. 2.3.5 program was used to determine the most probable number of genetic clusters within the population using Bayesian clustering and admixture model (individuals are allowed to have ancestry from multiple populations) [44]. Each individual isolate was allocated to one of the K numbers of populations. Five independent runs were accomplished for each K value to estimate the most probable number of genetic clusters with 500,000 Markov chain Monte Carlo (MCMC) iterations followed by a burn-in period of 100,000 iterations. The optimal number of genetic clusters was determined using the highest value of the likelihood, ln P(D), using Evano et al. [45] as implemented in Structure Harvester [46].

Allele frequency, expected heterozygosity (*He*) (defined as the probability that a pair of alleles randomly obtained from the population differs) [47] and relative haplotype frequency were estimated using ARLEQUIN v. 3.5 [48]. A minimum spanning network (MSN) was generated using *poppr* ver. 2.6.1, in R package, which is designed for analysis of populations with mixed modes of sexual and clonal reproduction [49]. The graphic software Inkscape (http://www.inkscape.org/) was employed to edit the network. Each haplotype was assigned a color code resembling the STRUCTURE output.

### 4.3. Detection of Recombination

To identify potential signatures of recombination, the index of association (*I_A_*) and standardized index of association $\left(\bar{r_{d}}\right)$ tests were computed for all the isolates as well as with clone-corrected data. The $\bar{r_{d}}$ test has the benefit over classical *I_A_* test, since it does not depend on the number of loci examined. *I_A_* is not significantly different from zero when the population is in linkage equilibrium (random association). Hypothesis testing was done with 1000 randomizations of the data set using Multilocus v.13 [50]. Further, *I_A_* analysis was performed with isolates grouped into genetic clusters based on the Bayesian clustering analysis and its clone corrected data with 1000 randomizations. In addition, pair-wise comparisons of multilocus linkage disequilibrium were also calculated for the eight microsatellite loci for all isolates as well as clone corrected data. GenAlEx 6.5 [51] was used to execute a principal coordinate analysis (PCoA) for clustering the population using microsatellite data.

### 4.4. Distribution of Mating Type Genes

Presence of inversion in the mating type loci (*MAT*) of *S. sclerotiorum* was first reported by Chitrampalam et al. [32] and with a simple PCR test the presence of Inversion-minus (Inv-) and inversion-plus (Inv+) isolates can be recognized. Inv- isolates show PCR products of both mating type idiomorphs, whereas Inv+ isolates show the PCR product of only the *MAT 1-2* idiomorph due to truncation of the *MAT 1-1* gene. Presence and absence of the *MAT* inversion in Sri Lankan population was assessed using two sets of PCR primers [11,32]. Amplifications were conducted in a 96 well thermal cycler (Applied Biosystem, USA) programmed as follows; initial denaturation at 95 °C for 8 min, 40 cycles of denaturation at 95 °C for 30 seconds, annealing at 55 °C for 30 seconds, extension at 72 °C for 60 seconds and final extension at 72 °C for 10 minutes. Nuclease free water was used as the negative control. PCR products were separated on 1% agarose gel for 45 min. to determine the PCR product sizes.

### 4.5. Detection of Multiple Infections per Cabbage Head and Detached Leaf Assay with Multiple Genotypes

MCG analysis was conducted for the 80 isolates collected from the three infection foci as described in Section 4.1. Isolates within a head and among heads in each infection focus were paired with one another in all possible combinations in this assay. It was presumed that a single MCG is present on the cabbage heads of a single infection focus due to rapid clonal reproduction and lack of multiple infections.

Fully expanded leaves of nine-week old cabbage variety “Green Corn” (obtained from regional agriculture research and development center, Bandarawela, Sri Lanka) were used for the detached leaf assay. Fully expanded leaves from the third node from the top were harvested and surface sterilized with 70% ethanol and washed with sterilized distilled water. Excess water from the leaf surface was removed with sterilized filter papers. Five isolates (isolates SS10, SS24, SS25, SS33, and SS36) belonging to four different MCGs (also four different MLHs) were cultured on water agar. Isolates SS10 and SS25 belonged to the same MCG (also from the same MLH) were also included for comparison purpose. Mycelial plugs (5 mm diam.) from the edge of actively growing three-day old colonies of one genotype were inoculated on one side of the leaf and the other side (0.5 cm away from the midrib from each side) was inoculated with a different genotype. Isolates of the same genotype on the same leaf served as controls. Three replicates were setup for each isolate combination. In total there were nine treatments in four groups; group 1 consisted of isolates SS10 and SS25 in three combinations as SS10 x SS10, SS10 x SS25, and SS25 x SS25; group 2 consisted of isolates SS25 and SS36 in three combinations as S25 x SS25, SS25 x SS36, and SS36 x SS36; group 3 consisted of isolates SS33 and SS36 in three combinations as SS33 x SS33, SS33 x SS36, and SS36 x SS36 and the last group consisted of isolates SS33 and SS24 in three combinations as SS33 x SS33, SS33 x SS24, and SS24 x SS24. Once inoculated, leaves were kept in a moist chamber and incubated at 23 °C in the dark. Photographs were taken two days after incubation and lesion area was measured using the software Image J (version 1.38) (http://rsb.info.nih.gov/ij/). Significant difference in lesion areas of the leaves inoculated with the same MCG and different MCGs were tested using one-way ANOVA and Tukey’s pairwise comparison.

## Figures and Tables

**Table 1:** Origin of *Sclerotinia sclerotiorum* isolates, their corresponding mycelial compatibility groups (MGCs) and multilocus haplotypes (MLHs).

Isolate Name ^a^	Location ^a^	MCG	MLH	Isolate Name ^a^	Location ^a^	MCG	MLH
SS5	Pattipola Field 2	1	1	SS11	Pattipola Field 4	3	2
SS26	Ambewela Field 1	1	1	SS15	Pattipola Field 5	3	2
SS35	Ambewela Field 3	1	1	SS19	Pattipola Field 6	3	2
SS36	Ambewela Field 4	1	1	SS31	Ambewela Field 1	3	2
A25	Seetha Eliya Field 5	1	1	A20	Seetha Eliya Field 3	3	2
A27	Seetha Eliya Field 6	1	1	SS18	Pattipola Field 6	3	15
G2	Meepilimana Filed 1	1	3	A15	Seetha Eliya Field 1	4	3
SS6	Pattipola Field 2	1	4	A26	Seetha Eliya Field 6	4	3
SS21	Pattipola Field 7	1	7	G1	Meepilimana Filed 1	4	3
A21	Seetha Eliya Field 3	1	7	G3	Meepilimana Filed 1	4	3
A16	Seetha Eliya Field 1	1	8	SS17	Pattipola Field 5	4	14
A22	Seetha Eliya Field 3	1	8	SS20	Pattipola Field 6	4	16
SS9	Pattipola Field 3	1	12	SS24	Pattipola Field 7	4	17
SS32	Ambewela Field 2	1	21	SS2	Pattipola Field 1	5	4
SS8	Pattipola Field 3	2	4	SS4	Pattipola Field 1	6	6
A23	Seetha Eliya Field 4	2	4	A18	Seetha Eliya Field 2	7	6
SS10	Pattipola Field 3	2	5	A19	Seetha Eliya Field 2	8	6
SS13	Pattipola Field 4	2	5	A24	Seetha Eliya Field 4	9	9
SS22	Pattipola Field 7	2	5	A17	Seetha Eliya Field 1	10	10
SS25	Pattipola Field 8	2	5	SS27	Ambewela Field 1	11	18
SS3	Pattipola Field 1	2	11	SS29	Ambewela Field 2	12	20
SS14	Pattipola Field 5	2	13	SS33	Ambewela Field 3	13	22
SS28	Ambewela Field 1	2	19	SS34	Ambewela Field 3	14	23
SS7	Pattipola Field 2	3	2				

^a^ Isolate names and locations are ordered according to mycelial compatibility groups (MCGs).

**Table 2:** Microsatellite loci, allele size, allele frequency and expected heterozygosity of the *Sclerotinia sclerotiorum* population.

Microsatellite Locus ^a^	Allele (bp) ^b^	Frequency ^c^	Expected Heterozygosity (*He*) ^c^
5-2	340	0.2340	0.4947
	342	0.6596	
	344	0.0638	
	346	0.0213	
7-2	180	0.0426	0.6957
	182	0.2553	
	190	0.4043	
	194	0.2979	
12-2	235	0.2340	0.6818
	237	0.3617	
	241	0.3830	
	243	0.0213	
13-2	313	0.0426	0.5402
	315	0.6596	
	329	0.1277	
	331	0.1277	
	333	0.0426	
17-3	375	0.0213	0.7438
	378	0.0426	
	381	0.4043	
	384	0.2553	
	399	0.1915	
	402	0.0638	
	405	0.0213	
55-4	176	0.9574	0.0842
	188	0.0213	
	204	0.0213	
110-4	392	0.2553	0.3885
	396	0.7447	
114-4	359	0.0426	0.8343
	383	0.1489	
	387	0.0213	
	395	0.2553	
	411	0.0213	
	415	0.2128	
	419	0.0213	
	435	0.1702	
	439	0.0638	

^a^ Eight microsatellite loci used in this experiment as described in Sirjusingh and Kohn [31]. ^b^ Allele size included M13 tail sequence and microsatellite repeat motifs. ^c^ Allele frequencies and expected heterozygosities were calculated using ARLEQUIN v.3.5.

**Table 3:** Comparison of genetic diversity of the pathogen population on cabbage with previously published two *Sclerotinia sclerotiorum* pathogen populations on canola.

	Host (Number of Samples)		
Genetic Diversity	Cabbage (N = 47)	Canola in China ^a^ (N = 30)	Canola in the USA ^a^ (N = 29)
**Gene diversity**			
Number of polymorphic loci	8	7	8
Total number of alleles	38	24	29
Mean number of alleles per locus	4.75	3	3.63
Mean expected heterozygosity (*He*)	0.56	0.46	0.59
**Genotypic diversity**			
Number of mycelial compatibility groups	14	27	19
Number of multilocus haplotypes (g)	23	29	19
Genotypic richness (g/N)	0.49	0.97	0.66

^a^ Results from Attanayake et al. [30] where the same eight microsatellite markers were used.

**Table 4:** Index of association (*I_A_*), standardized index of association ($\bar{r_{d}}$) values and their statistical significance for the whole population of *Sclerotinia sclerotiorum* and genetic clusters inferred from STRUCTURE analysis.

Population (N = Number of Isolates)	*I_A_*	$\bar{r_{d}}$	*p*
Whole population (N = 47)	1.524	0.224	<0.001
Clone corrected whole population (N = 23)	1.126	0.162	<0.001
Cluster 1 (N = 12) ^a^	0.036	0.018	0.463
Clone corrected cluster 1 (N = 6) ^a^	−0.455	−0.228	1.000
Cluster 2 (N = 12) ^a^	0.700	0.358	0.021
Clone corrected cluster 2 (N = 5) ^a^	0.771	0.387	0.13
Cluster 3 (N = 23) ^a^	1.661	0.279	<0.001
Clone corrected cluster 3 (N = 12) ^a^	1.211	0.202	0.001

^a^ Genetic clusters inferred from STRUCTURE analysis. Clusters 1, 2, and 3 are depicted in red, green and blue respectively in the Figure 1.

**Table 5:** Statistical significance (*p* values) of the pair-wise linkage disequilibrium tests for eight microsatellite loci of the clone corrected population of *Sclerotinia sclerotiorum*.

Locus	5-2	7-2	12-2	13-2	17-3	55-4	110-4
**7**-**2**	0.892						
**12**-**2**	0.972	1.000					
**13**-**2**	0.811	1.000	1.000				
**17**-**3**	0.947	1.000	1.000	1.000			
**55-4**	0.738	1.000	1.000	1.000	1.000		
**110**-**4**	1.000	1.000	1.000	1.000	1.000	1.000	
**114**-**4**	0.453	0.543	0.423	0.675	0.321	0.826	0.714
